# Microstructure and Superconductivity of Mechanically Alloyed Nb_0.67_(TiZrHf)_0.33_ High-Entropy Alloy

**DOI:** 10.3390/ma19112321

**Published:** 2026-05-31

**Authors:** Piotr Sobota, Wojciech Bartz

**Affiliations:** 1 Institute of Experimental Physics, University of Wrocław, pl. M. Borna 9, 50-204 Wrocław, Poland; 2 Institute of Geological Sciences, University of Wrocław, ul. Cybulskiego 30, 50-205 Wrocław, Poland; wojciech.bartz@uwr.edu.pl

**Keywords:** high-entropy alloy, superconductor, mechanical alloying, microstructure

## Abstract

A superconducting high-entropy alloy (HEA) Nb_0.67_(TiZrHf)_0.33_ powder was successfully synthesized via mechanical alloying for the first time. X-ray diffraction, scanning electron microscopy, energy dispersive X-ray spectroscopy, magnetic measurements, and specific heat were used to investigate its structural and physical properties. The alloy was crystallized in a single-phase body-centered cubic structure with a small amount of non-magnetic impurities coming from ball milling. Specific heat data confirms the presence of bulk superconductivity in the as-synthesized state, with the broadness of the thermodynamic anomaly reflecting the significant chemical disorder and distribution of critical temperatures typical of HEAs. $T_{c}$ is in the range 6–7.5 K, and the upper critical field $\mu 0H_{c2}$ is in the range 6.4–7.6 T. These results demonstrate that mechanical synthesis is a viable route for producing superconducting HEA powders, which are promising candidates for consolidation via sintering and provide a robust platform for investigating superconductivity in highly disordered systems.

## 1. Introduction

High-entropy alloys (HEAs) can be defined as homogeneous solid solutions of multiple elements mixed in non-negligible proportions, exhibiting the “HEA core effects” as described by Yeh et al. [1]. Despite the fact that they are formed from multiple mixed elements, they retain uniform, simple, and dense structures (most often BCC, FCC, and HCP). As the main advantage of HEAs over regular alloys is their mechanical [2] and chemical resistance [3], they have attracted the attention of many research teams and have been extensively investigated. Superconducting high-entropy alloys have seen significant development in the past few years, aiming to discover new systems with increasingly superior critical parameters [4]. Apart from the pristine composition originally reported by P. Kozelj et al. in 2014 [5], a number of different materials have been explored. Among them are other BCC-structured systems, such as Nb-Ta-based alloys [6,7,8] and high-$H_{c2}$ Ti-rich alloys [9,10]. Additionally, a recent report even showed a BCC Cu-containing HEA superconductor [11]. Furthermore, systems containing actinides have attracted attention due to the anomalous behavior of their upper critical field [12]. In the case of BCC HEA alloys, another interesting property was observed: their critical temperatures seem to violate the Matthias rule regarding the Valence Electron Count [13]. Although the majority of well-investigated systems are superconducting HEAs with a BCC structure [14,15,16], there is also a significant and growing number of HEAs crystallizing in other systems, such as FCC [17], sigma [18], A15 [19], α-Mn [20], and HCP structures [21]. Nonetheless, the number of accessible synthesis routes has also increased, which has allowed for more alloys to be discovered and has enabled previously obtained ones to be characterized with different properties. Such examples can be seen in recent papers highlighting the differences between materials obtained in the form of either powders [22,23] or thin layers [24,25] compared to the bulk form. This is particularly interesting in the context of the potential use of high-entropy materials in superconducting devices, such as superconducting wires and magnets. Especially because, due to the HEA mechanical properties, machining of the cast alloys has proven to be difficult.

Taking advantage of high-entropy effects and intrinsic internal disorder can yield exceptional results in critical current densities. In this work, we report the results on a mechanically alloyed Nb_67_(TiZrHf)_33_ alloy, which was previously reported [6,26], although without extensive details regarding its synthesis or superconducting parameters beyond $T_{c}$. There is generally little information available on superconducting HEAs synthesized directly in powder form. The niobium-rich matrix of this chemical composition, when combined with more brittle elements such as Zr and Hf, suggested that this alloy could be synthesized via mechanical alloying directly into a state exhibiting bulk superconductivity, unlike similar compositions in previous reports [23]. The sample underwent structural characterization by XRD and morphology assessment by SEM-EDXS. Both methods show a very uniform, homogeneous structure. In contrast, magnetic and specific heat investigations underscore the superconducting state behavior, showing significant inhomogeneity. This raises questions regarding the reliability of standard techniques for assessing sample quality in multicomponent alloys, especially those formed from refractory metals.

## 2. Materials and Methods

Polycrystalline sample of Nb_0.67_(TiZrHf)_0.33_ was synthesized using the mechanical alloying method, using the planetary ball mill Pulverisette 6 classic line. The raw, commercial (Alfa Aesar, Heysham, UK) powders of Nb, Ti, Zr, and Hf, of at least 99.9% purity and mesh size of 325 (below 44 μm size), were placed in appropriate proportions in an 80 mL tungsten carbide (WC) vial with WC balls. The powders’ mass was 12 g, and the ball-to-powder mass ratio was ∼16:1. The vial was then sealed and flushed several times with 4N argon gas to remove air and was left with an Ar pressure about 2 bar above atmospheric to prevent air diffusion through rubber seals. Then, the ball milling was performed at 250 RPM with a total active milling time of 20 h, during which for every 12 min of active milling there was 30 min of rest to prevent overheating and oxidation; so, the whole process took approximately 65 h. After the synthesis, the vial was resting for 24 h before it was opened with an overpressure of Ar still present inside, indicating good oxidation protection. The powder was then partially removed from the vial walls using a hardened steel tool. The total yield of the synthesis was ∼40% of mass, which was influenced by the use of Nb which is a malleable metal and the fact that no other method of powder extraction was used to protect the supposed uniformity of the sample morphology.

The morphology, chemical composition, and homogeneity of the obtained sample was verified by energy dispersive X-ray spectroscopy (EDXS) using a JEOL JSM-IT100 In-Touch-Scope (Japan)^TM^ scanning electron microscope. The crystal structures of the powder sample was studied by powder X-ray diffraction (XRD) using a PANalytical X’pert Pro diffractometer (UK) with CuKα radiation. The experimental XRD pattern was analyzed using the Rietveld method using FULLPROF software, ver. febuary 2025 [27].

The magnetic properties of the powder alloy were studied in the temperature range of 1.8 K to 8 K and in magnetic fields up to 70 kOe using a MPMSII option in the commercial Quantum Design DynaCool Physical Properties Measurement System. The heat capacity was measured from room temperature to 1.8 K and in fields up to 70 kOe using a DynaCool platform.

## 3. Results and Discussion

### 3.1. Crystal Structure

**Figure 1:**
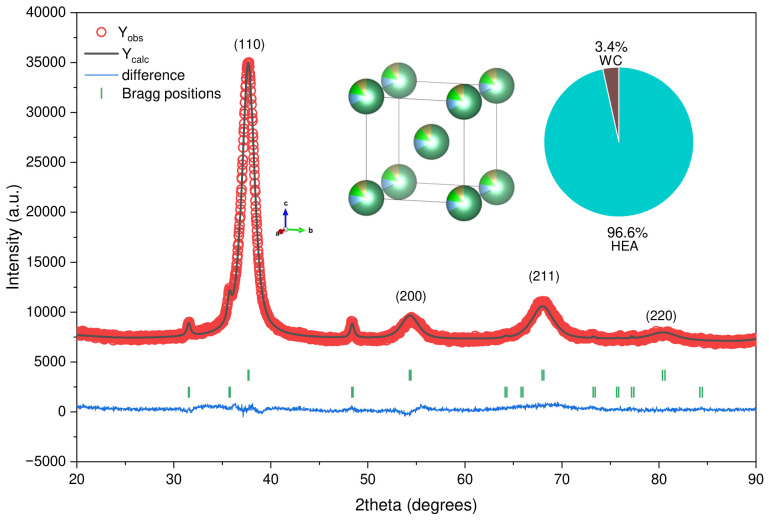
X-ray diffraction patterns and results of Rietveld refinements of Nb_0.67_(TiZrHf)_0.33_. The red circles and gray line represent the experimental points and the theoretical curve, respectively. The blue line shows the difference between the two, and green vertical dashes indicate the positions of the Bragg reflections for observed phases. The upper lines are referencing BCC HEA structure while lower the WC hexagonal structure. The presented structure visualization is referencing the HEA phase, while the pie chart shows the % of both of the phases in the studied sample based on the results of the Rietvled refinement.

The X-ray diffraction pattern for the obtained alloy is presented in Figure 1. The analysis by the Rietveld method of the experimental data showed that sample obtained by mechanical alloying consisted of two phases. The major phase (96.6 at.% according to the Rietvled refinement) was an HEA Nb_0.67_(TiZrHf)_0.33_ phase with the space group $Im\bar{3}m$, a W-type structure, and *a* = 3.376(1) Å. The other, minor, hexagonal phase with *a* = 2.902(1) Å and *b* = 2.836(1) Å was ascribed to tungsten carbide (WC), which is a contamination from high-energy ball milling. The resulting fit was of good quality, which was indicated by the reliability factors (not corrected for the background): R_*p*_ = 1.81, and R_*wp*_ = 2.30.

In fact a contamination from the vial or balls is very common, while regular vials are made from steel they poses a risk of contaminating material with iron or other magnetic metals. On the other hand, process controlled agents like stearic acid or other surfactants can lead to the formation of the magnetic carbide impurities. Tungsten carbide, as a magnetically inert material, does not introduce pair-breaking effects that would interfere significantly with the magnetic signal of the superconductor and is considered non-reactive at ambient temperatures.

Utilizing the calculated diffraction peak from Rietveld refinement of the obtained XRD pattern, a linear fit was made according to the Williamson–Hall formula [28]:

(1) $$\beta \cos\theta =\epsilon \sin\theta +\frac{K\lambda }{L}.$$

In this equation β represents the full width at half maximum (FWHM) of the intensity in 2θ units, θ is the Bragg angle, λ is the X-ray wavelength, and *K* = 0.9 is the approximate shape factor. The main HEA phase had a low average crystalline size *L* = 21(1) nm and high average lattice strain Cϵ = 5.69(1) %.

### 3.2. Morphology and Homogeneity

**Figure 2:**
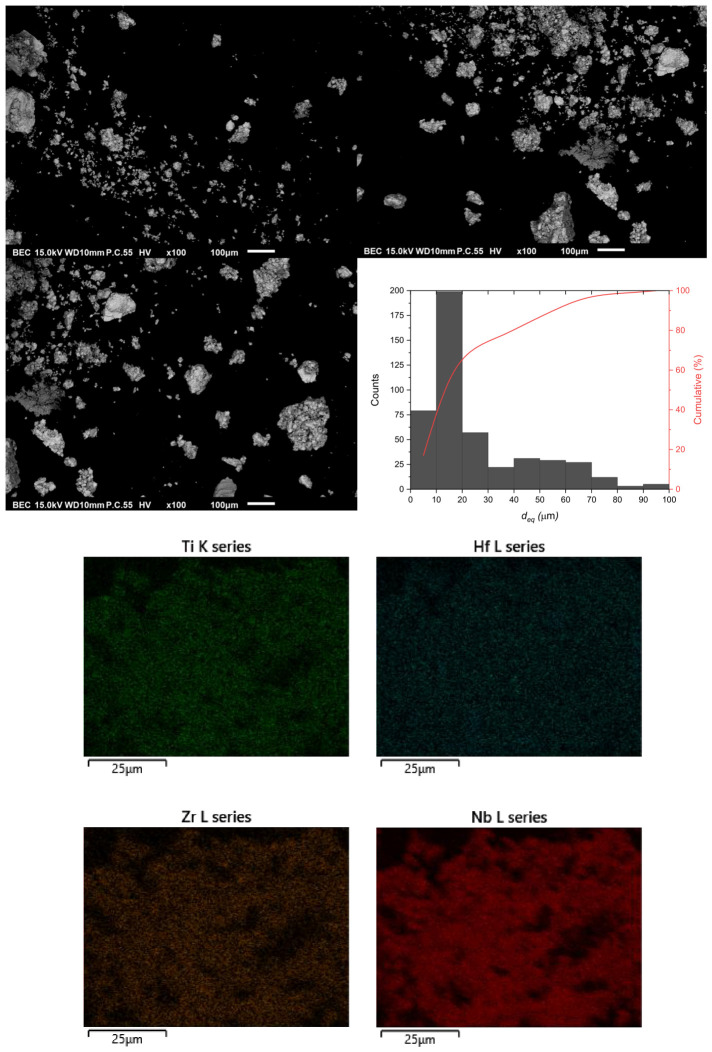
The SEM micrographs of the Nb_0.67_(TiZrHf)_0.33_ powders with EDXS maps superimposed on them. The histogram shows the grain size distribution of the agglomerates.

The representative SEM micrographs of the Nb_0.67_(TiZrHf)_0.33_ alloy are presented in Figure 2. They reveal that the powder consists of rather soft agglomerates, with no individual crystallites visible at this scale. Particle size analysis performed using ImageJ 13.0.6. provided a dataset of their surface areas. Due to the irregular morphology of the agglomerates resulting from mechanosynthesis, the Equivalent Circular Diameter (ECD) approximation was applied:

(2) $$d_{eq}=\sqrt{\frac{4A}{\pi }},$$

where $d_{eq}$ is ECD, and *A* is agglomerate area. The results are illustrated as a histogram in Figure 2. The distribution indicates that the dominant agglomerate size ranges between 10 and 20 μm.

The EDXS maps show good uniformity of the material with homogeneous element distribution. Based on the point EDXS measurements, the element content (in at.%) was as follows: Nb-66.2(5); Hf-11.9(4); Zr-11.6(2); and Ti-10.3(1), which is in good agreement with the nominal values (67 for Nb and 11 for other elements). While EDXS data alone is highly resolution-dependent, its combination with XRD provides a solid foundation for structural and chemical homogeneity, which is ultimately confronted with the superconducting properties discussed later in this paper.

However, on the individual crystalline scale, homogenity may not necessarily be conserved. Taking into account that both the nature of the high-entropy alloys and the method of synthesis promote random distribution of the elements in the lattice, there can still exist large chemical and structural disorder inside individual crystallites. Especially because their size is on the order of 20 nm, there can exist deviations from exact stoichiometry, and diverse local atomic environments can be formed. Such deviations may be clearly evident when measuring the superconducting properties of the alloy, particularly its specific heat anomaly and AC magnetic susceptibility.

#### 3.2.1. Magnetic Properties

**Figure 3:**
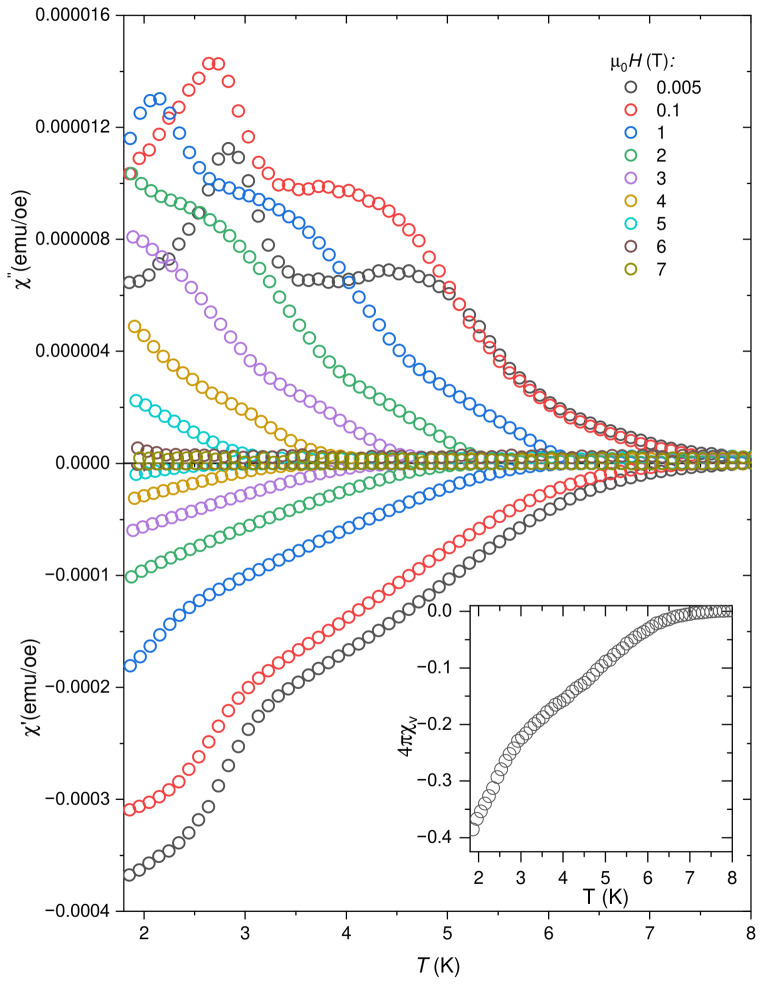
Real $\chi^{\prime }$ and imaginary $\chi^{\prime \prime }$ parts of AC susceptibility of the Nb_0.67_(TiZrHf)_0.33_ powders measured in the nominal applied field up to 7 T. The insert shows the dimensionless susceptibility based on the DC measurement in a 0.005 T applied field.

Figure 3 shows the results of the AC susceptibility measurements of Nb_0.67_(TiZrHf)_0.33_ powder alloy. The real part ($\chi^{\prime }$) demonstrates a very broad diamagnetic superconducting transition starting around 7 to 8 K, which is consistent with a material possessing a highly deformed crystal structure (as indicated by the Rietveld analysis) induced by mechanical alloying. The inset shows the volume susceptibility ($4\pi \chi_{v}$), derived from the DC measurements, reaching only about −0.4 at 1.8 K, rather than the ideal −1 expected for perfect diamagnetism. This can indicate a reduced superconducting volume fraction. This can be caused by several parallel factors: the macroscopic non-magnetic tungsten carbide (WC) admixture introduced by the milling process [29], the high density of non-superconducting defect-rich regions on the heavily deformed grains, and the highly expanded body-to-surface ratio, especially in the smallest agglomerates [30]. The latter can be also affected by some degree of surface oxidation. Collectively, these factors lead to a reduction in the superconducting volume fraction in relation to the samples mass, thereby decreasing the dimensionless susceptibility ($4\pi \chi_{v}$).

The imaginary part ($\chi^{\prime \prime }$) exhibits energy dissipation peaks associated with magnetic flux dynamics. These dissipation peaks broaden considerably and shift toward lower temperatures as the applied DC magnetic field ($\mu_{0}$H) increases from 5 mT to 7 T. This field-dependent behavior, together with the strongly smeared transitions, is characteristic of a highly disordered type-II superconductor with the extensive network of structural defects generated during ball milling. It can also indicate the presence of strong but complex flux pinning centers.

In the real part of the susceptibility ($\chi^{\prime }$), the diamagnetic screening exhibits a noticeable “two-step” drop or a kink rather than a single smooth curve. It is visible for the lowest applied field, below 1 T. Also, the imaginary part ($\chi^{\prime \prime }$) shows a complex structure that suggests a secondary shoulder or a merged twin-peak at those two low fields. Given that the XRD results confirmed a pure (though highly deformed) superconducting bcc phase, this two-step feature can be mainly explained by two phenomena. First, it can be an indicator that the material exhibits granular superconductivity [31,32]. If that would be correct, the higher-temperature peak would represent the intragranular transition, where the relatively intact cores of the grains become superconducting. The lower-temperature step would reflect the intergranular coupling and represent the point at which the superconducting phase manages to establish weak links across the heavily deformed, defect-rich, and possibly non-superconducting grain boundaries. However, the SEM observations failed to detect at least a significant number of large intact grains that would corroborate this interpretation.

Second, given the nature of high-entropy alloys, there is also another possible explanation. The above-mentioned high-degree of chemical disorder coupled with the small size of the crystalline phase can produce two chemically distinct phases that are structurally very similar yet their superconducting properties, especially the critical parameters, would be different. Such inhomogenity would be particularity visible in specific heat measurements and has been observed in HEA superconductors produced by both mechanical alloying and by arc melting [23,33].

**Figure 4:**
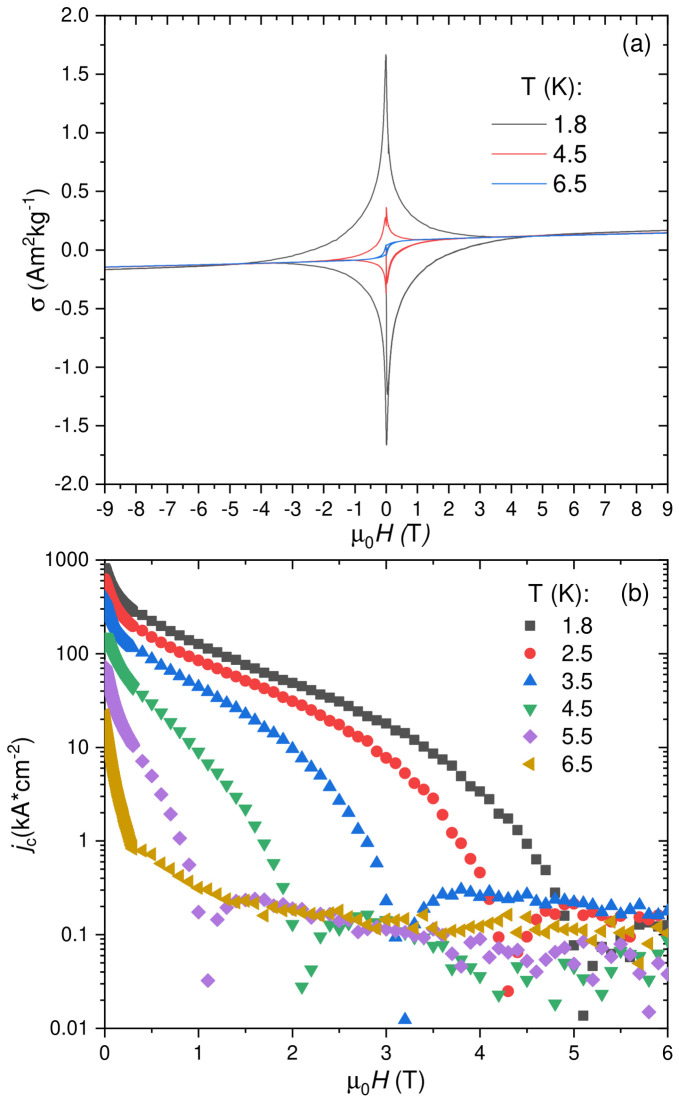
The magnetic hysteresis loops from the selected temperatures (**a**) and critical current densities (**b**) of the Nb_0.67_(TiZrHf)_0.33_ powder.

Figure 4a presents the sets of five quarts of magnetization isotherms of the Nb_0.67_(TiZrHf)_0.33_ alloy displaying broad loops characteristic of strong magnetic pinning and with clearly visible irreversible fields values. The loops are strongly temperature dependent showing that the thermal fluctuations can easily unpin the magnetic vortices in this material. Additionally, at higher fields, the magnetization does not perfectly plateau but exhibits a slight upward slope, suggesting a weak paramagnetic background likely originating from tungsten carbides or amorphous oxides formed at the surface of the powders. In Figure 4b critical current density ($j_{c}$) curves are presented. They were calculate based on the Bean model [34], approximating the spherical shape of the crystallines:

(3) $$j_{c}=\frac{3\Delta Md}{R}$$

where ΔM is the broadness of hysteresis loop, *d* is the density of the material, and R is the sphere radius, which is the same as the dominant value of $d_{eq}$ from Equation (2). The maximal value of $j_{c}$ (1.8 K) is around 800 kA cm^−2^ which confirms strong pinning, but as the applied magnetic field ($\mu_{0}H$) increases, $j_{c}$ experiences a sharp, exponential drop which points to the presence of weak links at the heavily deformed, defect-ridden grain boundaries, where intergranular phase coherence is easily destroyed by the penetrating magnetic field. Despite several attempts, the analysis of the flux pinning mechanism using the established Dew–Hughes [35] and Griessen [36] models proved unsuccessful. This difficulty most likely arises from the coexistence of two distinct superconducting phases within the material, which leads to the interpretation that a substantial degree of chemical disorder is present within the crystallites themselves.

#### 3.2.2. Specific Heat

**Figure 5:**
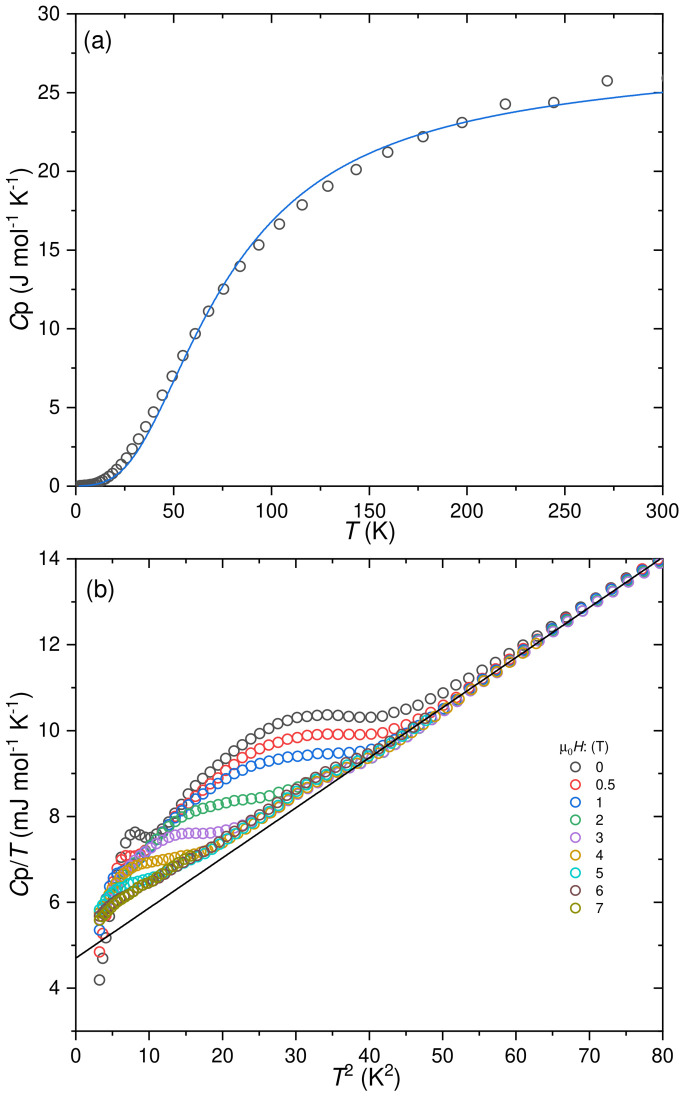
Temperature variation of specific heat $C_{P}$ of Nb_0.67_(TiZrHf)_0.33_ measured in nominal zero magnetic field. The solid blue line shows a fit to the Debye Equation (4). (**a**) $C_{P}/T$ vs. $T^{2}$ measured in various external magnetic fields $\mu_{0}H$ for the Nb_0.67_(TiZrHf)_0.33_ sample. The straight solid line is a fit of Equation (5) to the experimental data (**b**).

The results of the measurements of the specific heat at the nominal zero field and temperatures from RT to 1.8 K are presented in Figure 5a. They demonstrate typical metallic behavior across the entire measured temperature range. Aside from the low-temperature superconducting anomaly, the $C_{p}\left(T\right)$ curve is featureless and can be accurately modeled using the standard Debye formula:

(4) $$C_{p}\left(T\right)=\gamma T+9Rr{\left(\frac{T}{\Theta_{D}}\right)}^{3}\int_{0}^{\Theta_{D}/T}\frac{x^{4}e^{x}}{{\left(e^{x}-1\right)}^{2}}dx,$$

with the resulting Debye temperature of 304(4) K. At high temperatures, the specific heat capacity ($C_{p}$) steadily increases and flattens out, closely approaching the Dulong–Petit limit of approximately 25 J mol^−1^∗K^−1^, which indicates that all lattice vibrational phonon modes are fully excited, and there are no significant deviations from formal composition.

Figure 5b shows the low temperature part of the specific heat measured in magnetic fields up to 7 T plotted as $C_{p}/T$ versus $T^{2}$. The $C_{p}/T$($T^{2}$) at low temperatures can be described as a sum of heat contribution (connected with γ coefficient) and the phonon heat contribution (with the corresponding β coefficient) The solid black curves represent the fits of the Equation (5) to the experimental data. The resulting parameters from the fit are as follows: γ = 4.71(8) mJ K^−2^ mol^−1^, and β = 0.117(1) mJ K^−4^ mol^−1^.

(5) $$\frac{C_{P}\left(T\right)}{T}=\gamma +\beta T^{2},$$

At zero applied magnetic field, there is a thermodynamic anomaly showing the bulk transition into the superconducting state. This jump, however, is noticeably broad and smeared rather than a sharp λ-type transition. Because specific heat is a bulk volume measurement, this complex shape points to mesoscopic phase separation. It suggests that the mechanical synthesis introduced severe chemical and/or local structural disorder, separating the seemingly pure bcc phase (as observed by the XRD) into distinct superconducting fractions with slightly different critical temperatures ($T_{c}$). As the external magnetic field is progressively increased, this superconducting anomaly is systematically suppressed in amplitude and shifted towards lower temperatures. At zero field and low temperature, the second peak can be observed at low field and eventually gets suppressed by the increasing magnetic field. The value of the $T_{c}$ onset generally matches the values extracted from magnetic measurements, but due to broadening and the presence of the second peak, it is impossible to correctly establish critical temperature (as the mid point between normal and superconducting phase) using classical equal entropy construction.

Taking the calculated value of the coefficient β and the relation Equation (6) (where R is universal gas constant and r = 1), the Debye $\Theta_{D}$ temperature was estimated at 255(2) K, significantly lower than the temperature estimated from the entire temperature range.

(6) $$\beta =\frac{12}{5}rR\pi^{4}{\left(\frac{T}{\Theta_{D}}\right)}^{3},$$

Using the onset of $T_{c}$ (estimated as 7 K) and the $\Theta_{D}$ value, the electron-phonon coupling constant was calculated using McMillan’s equation [37]

(7) $$\lambda_{\mathrm{el}-\mathrm{ph}}=\frac{1.04+\mu^{*}\ln\left(\frac{\Theta_{D}}{1.45T_{c}}\right)}{\left(1-0.62\mu^{*}\right)\ln\left(\frac{\Theta_{D}}{1.45T_{c}}\right)-1.04},$$

where $\mu^{*}$ is the Coulomb repulsion constant (assumed as 0.125, a value commonly used for *d*-electron systems). The obtained value of $\lambda_{\mathrm{el}-\mathrm{ph}}$ was 0.75, which places this alloy in the weak-to-intermediate coupling range. Assuming $\Theta_{D}$ from Equation (4), the value of $\lambda_{\mathrm{el}-\mathrm{ph}}$ would be 0.7, which places the alloy in the same coupling range.

Utilizing the relation

(8) $$\gamma =\frac{1}{3}\pi^{2}k_{B}^{2}N_{A}N\left(E_{F}\right)$$

where $k_{B}$ is the Boltzmann constant, and $N_{A}$ is the Avogadro number, the experimental density of states of conduction electrons at the Fermi level $N(E_{F})$ were estimated as 2 states eV^−1^ f.u.^−1^. At the same time, the densities of non-interacting electrons $N{\left(E_{F}\right)}^{*}$ were estimated as 1.3 states eV^−1^ f.u.^−1^, using the relation [38]:

(9) $$N{\left(E_{F}\right)}^{*}=\frac{N(E_{F})}{1+\lambda_{\mathrm{el}-\mathrm{ph}}}.$$

Also, the Pauli limiting field $\mu_{0}H_{P}$ = 12.9 T was calculated from the relation [39]:

(10) $$\mu_{0}H_{P}=1.84T_{c}.$$

**Figure 6:**
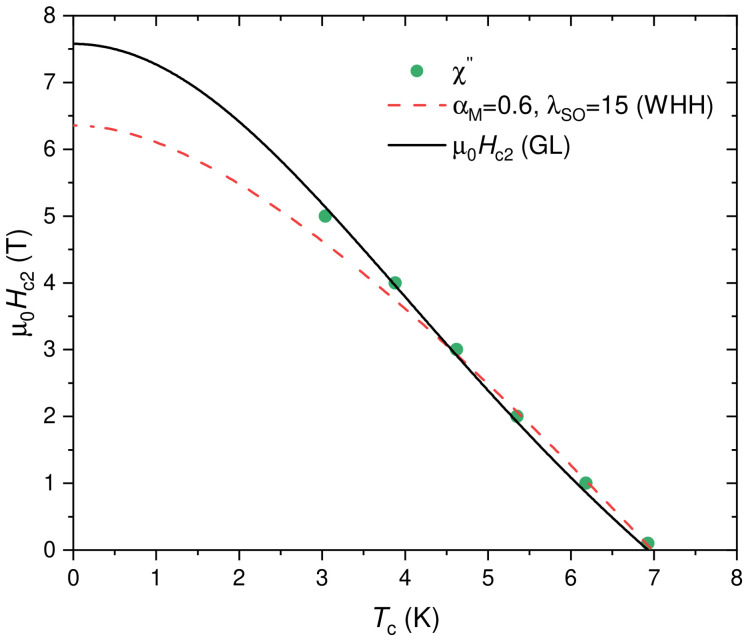
Upper critical field of Nb_0.67_(TiZrHf)_0.33_ as a function of temperature and derived from AC susceptibility data. The dashed red line is the simulated WHH curve (Equation (12)), while the solid black line is a fit to the Equation (11).

The estimation of the upper critical field was based on the onset of $T_{c}$ in the imaginary (χ″) part of the AC susceptibility instead of relying on direct $C_{p}$ data, because it yielded the sharpest and most precisely determinable transition onset for all applied magnetic fields. The data and results of the applied fitting model are presented in Figure 6.

The $\mu_{0}H_{c2}$($T_{c}$) data can be described by the commonly used Ginzburg–Landau equation:

(11) $$\mu_{0}H_{c2}\left(T\right)=\mu_{0}H_{c2}\left(0\right)\frac{1-{\left(T/T_{c}\right)}^{2}}{1+{\left(T/T_{c}\right)}^{2}}$$

giving an upper critical field at 0 K 7.6(1) T. However, because the GL model is phenomenological and macroscopic, it tends to overestimate values of $\mu_{0}H_{c2}$ at low temperatures. Therefore to obtain a more correct assessment of the $\mu_{0}H_{c2}$, we applied the Werthamer–Helfand–Hohenberg (WHH) [40,41,42] model for BCS superconductors in the dirty limit. This model includes the spin-paramagnetic effect (described by the Maki parameter $\alpha_{M}$) and the spin-orbit scattering constant $\lambda_{\mathrm{SO}}$ to fit the experimental AC data (Equation (12), where $\gamma \equiv \sqrt{{\left(\alpha_{M}\bar{h}\right)}^{2}-{\left(\frac{1}{2}\lambda_{\mathrm{SO}}\right)}^{2}}$, $\bar{h}=\frac{4}{\pi^{2}}\frac{H_{c2}}{-dH_{c2}/dt}$, $t\;=\;\frac{T}{T_{C}}$, and ψ is a digamma function):

(12) $$\begin{array}{c} \;\ln\frac{1}{t}=\left(\frac{1}{2}+\frac{i\lambda_{\mathrm{SO}}}{4\gamma }\right)\psi \left(\frac{1}{2}+\frac{\bar{h}+\frac{1}{2}\lambda_{\mathrm{SO}}+i\gamma }{2t}\right)+\\ \;\left(\frac{1}{2}-\frac{i\lambda_{\mathrm{SO}}}{4\gamma }\right)\psi \left(\frac{1}{2}+\frac{\bar{h}+\frac{1}{2}\lambda_{\mathrm{SO}}-i\gamma }{2t}\right)-\psi \left(\frac{1}{2}\right)\; \end{array}$$

The calculated upper critical field $\mu_{0}H_{c2}\left(0\right)$ was found to be 6.4(1) T. The low Maki parameter ($\alpha_{M}=0.6$) indicates that the transition is governed primarily by orbital limiting rather than paramagnetic pair-breaking effects. At the same time, the high spin-orbit scattering parameter ($\lambda_{so}=15$) is consistent with the high level of structural disorder and chemical inhomogeneity introduced during mechanical alloying. While high $\lambda_{so}$ typically mitigates the Pauli limit, the overall moderate value of $\mu_{0}H_{c2}$ suggests that the extensive lattice deformation is the dominant factor defining the superconducting state’s field-dependent behavior.

It should be noted, however, that the chemical inhomogenity of the alloy, as confirmed by specific heat measurements, does not entirely rule out the possibility that the material is, to some extent, a granular superconductor, but it certainly shows that the nature of the HEA has a dominant influence on the superconducting state, and that magnetic measurements alone are not sufficient to determine this. Specifically, while magnetization data may merely reflect macroscopic shielding from coupled grains, the specific heat anomaly provides direct evidence for bulk superconductivity inherent to the heavily disordered HEA.

## 4. Conclusions

A superconducting high-entropy alloy (HEA) Nb_0.67_(TiZrHf)_0.33_ powder was successfully synthesized for the first time via mechanical alloying. The material crystallizes in a single-phase body-centered cubic (bcc) structure, containing only minor tungsten carbide (WC) impurities from the milling process. Microstructural analysis reveals a fine-grained morphology with high chemical homogeneity, as confirmed by SEM and EDXS. Magnetic measurements indicate a high degree of structural disorder and features characteristic of granular superconductivity, likely originating from the extensive deformation during synthesis. While the critical current density derived from the Bean model is high at low fields, it exhibits a rapid decay, pointing to a relatively low irreversibility field. Notably, bulk superconductivity is observed directly in the as-synthesized state. Specific heat results further emphasize the dominant HEA character, where significant chemical disorder leads to a broad distribution of local critical temperatures. Given its robust superconducting properties and resistance to deformation, this alloy represents a compelling platform for studying the nature of superconductivity in HEAs and remains highly promising for subsequent powder metallurgy processing, such as spark plasma sintering (SPS), for the powder-in-tube method (PIT). These two post-processing routes could enable the fabrication of robust superconducting magnets and wires operating at liquid helium temperatures.

## Data Availability

The original contributions presented in this study are included in the article. Further inquiries can be directed to the corresponding author.
